# Hydrolytic Hydrogen Production on Al–Sn–Zn Alloys Processed by High-Pressure Torsion

**DOI:** 10.3390/ma11071209

**Published:** 2018-07-13

**Authors:** Fan Zhang, Kaveh Edalati, Makoto Arita, Zenji Horita

**Affiliations:** 1 School of Materials Engineering, Shanghai University of Engineering Science, Shanghai 201620, China; 2 WPI, International Institute for Carbon-Neutral Energy Research (WPI-I2CNER), Kyushu University, Fukuoka 819-0395, Japan; kavedalati@gmail.com (K.E.); horita@zaiko.kyushu-u.ac.jp (Z.H.); 3 Department of Materials Science and Engineering, Faculty of Engineering, Kyushu University, Fukuoka 819-0395, Japan; arita@zaiko.kyushu-u.ac.jp

**Keywords:** hydrogen production, ultrafine-grained (UFG) structure, high-pressure torsion (HPT), severe plastic deformation (SPD), hydrolysis

## Abstract

Aluminium-tin-based alloys with different compositions were synthesized by a high-pressure torsion (HPT) method. The effect of different alloying elements and processing routes on the hydrogen generation performance of the alloys was investigated. The results show that Zn can enhance the hydrogen generation rate and yield by promoting pitting corrosion. The highest reactivity in water was achieved for an Al-30wt %Sn-10wt %Zn alloy. Detailed analysis of the Al-30wt %Sn-10wt %Zn alloy shows that increasing the shear strain and the resultant formation of ultrafine grains and phase mixing enhance the hydrogen generation rate through the effects of both nanogalvanic cells and pitting corrosion.

## 1. Introduction

Hydrogen is the most abundant element in the universe. As a fuel, it has a high calorific value and the highest energy density per unit mass. It can react with oxygen and produce water and energy. Because of these benefits, hydrogen is considered to be the most suitable clean-energy carrier to replace fossil fuels in future [1,2,3,4,5]. However, hydrogen is not freely available in nature and it must be produced from the decomposition of other chemicals such as hydrocarbons. Steam reforming of hydrocarbons (usually natural gas) is currently the main technique for hydrogen production, but this technique uses nonrenewable energy sources and produces large amounts of CO_2_ [4,5]. Water splitting is considered to be the most nature-friendly technique for hydrogen production [4].

Water is the most abundant source for hydrogen on earth. There are several techniques for water splitting and production of hydrogen [4]: (i) thermal splitting of water, which is a complex process and needs very high temperatures; (ii) water electrolysis, which can use solar energy as a source of electricity but is rather expensive; and (iii) photocatalytic hydrogen generation, which only uses a photocatalyst and solar energy, but the efficiency of the method is too low. Another technique to produce hydrogen is by reaction between water and scraps of alkali metals and Al (hydrolysis technique).

Among metals which can be used for hydrolysis, Al has received appreciable attention due to its abundance on the earth, high theoretical hydrogen production (1.5 mole H_2_ is produced from 1 mole Al), and simplicity of the production system [6,7,8]. However, the passivation and difficult activation of Al in neutral water remains a problem for Al/water reaction. So far, many methods have been developed to solve this activation problem, including the addition of alloying elements and the introduction of lattice strain through ball milling [9,10,11,12,13].

Bulk severe plastic deformation (SPD) techniques such as high-pressure torsion (HPT) can be employed for mechanical alloying and introduction of lattice strain and defects in different materials [14,15]. There has been a successful attempt to activate the Al/water reaction by SPD processing through the HPT method [16]. It was shown that the formation of nanogalvanic cells by the addition of Sn (which has a higher electrode potential than Al) and subsequent HPT processing can enhance the hydrogen generation rate. However, the reaction rate is still not sufficiently high and the total hydrogen production is much smaller than the expected theoretical level. Therefore, other strategies should be developed to enhance the reaction rate.

In this work, a third element is added to the Al–Sn alloys to promote the hydrolysis reaction by not only the formation of nanogalvanic cells but also by promoting pitting corrosion. Since copper, silicon, magnesium, and zinc are commercially added to Al alloys, and iron and nickel are usually present as impurities in the form of Al-based intermetallics, these elements were added to the Al–Sn system by mechanical alloying through the HPT process or by melting to enhance the hydrogen generation performance. The fractions of these elemental additives were optimized empirically to achieve the best hydrolytic hydrogen generation.

## 2. Materials and Methods

Different Al–Sn-based compositions such as Al-50wt %Sn-10wt %X (Zn, Cu, Ni, Mg Fe, Si), Al-*x*wt %Sn-10wt %Zn (*x* = 10, 20, 30, 40, 50), and Al-30wt %Sn-*x*wt %Zn (*x* = 5, 10, 20, 30) were prepared from elemental powders by mechanical agitation and processed using similar HPT processing conditions for *N* = 10 turns under *P* = 6 GPa at room temperature (~300 K). The purity and grain size of the starting powders are given in Table 1. In order to investigate the effect of processing (casting, cold rolling, and HPT processing) on the hydrogen generation performance, an Al–Sn–Zn ingot was prepared from pure Al (99.99%), Sn (99.99%), and Zn (99.99%), with an Al:Sn:Zn mass ratio of 6:3:1. As will be shown in the manuscript, this composition showed the best hydrogen generation performance among the all selected compositions. The melting process was carried out in a stainless steel crucible under the air atmosphere at 1073 K. The melt was stirred and kept at a high temperature until the alloying elements were dissolved in each other. The melt was then poured into a steel mould to form an ingot with dimensions of 30 × 30 × 50 mm^3^. The subsequent homogenization was performed in a muffle furnace at 483 K for 24 h. After homogenization, disc samples with 10 mm diameter and ~0.9 mm thickness were cut manually from the ingot using a rotary cutter and a 10-mm diameter punch for HPT processing. A plate with initial dimensions of 20 × 20 × 1.7 mm^3^ was also cut manually for cold rolling. Mixed powders with ~0.5 g were processed by HPT at room temperature under a pressure of *P* = 6 GPa. Shear strain was introduced through the rotation of the lower anvil with respect to the upper one for *N* = 1, 5, 10, and 20 turns with a rotation speed of 1 rpm. The plate with initial dimensions of 20 × 20 × 1.7 mm^3^ was rolled at room temperature until its thickness reached ~1 mm (rolling ratio: 40%).

Hydrogen generation was examined in a beaker containing 400 mL pure water with a resistivity of 18.2 MΩ cm (at 298 K) at a temperature in the range of 296–333 K using a water displacement method. All the samples were ground mechanically by emery papers to be an equal surface condition before being immersed in the water. For samples immersed into water at room temperature, the volume of generated hydrogen was recorded every day, while for the samples examined at elevated temperatures, the hydrogen volume was recorded every 5 min during the first hour and every 1 h during the subsequent 2 h.

A conventional three-electrode cell configuration was employed to perform the electrochemical measurements. A Pt spiral wire was used as the counter electrode, and an Ag/AgCl electrode saturated with KCl was used as the reference electrode. Open circuit potential (OCP) curves of samples were recorded for several hours after the immersion.

Vickers microhardness was measured with an applied load of 50 g for 15 s along the radii at 12 different radial directions to check the homogeneity along the radii. XRD analysis was performed using the Cu Kα radiation in a scanning step of 0.01° and a scanning speed of 1°/min. For transmission electron microscopy (TEM) and scanning transmission electron microscopy (STEM), a focused ion beam (FIB) system was used to prepare thin foils from the disc samples at ~4 mm away from the disc centre. TEM and STEM observation was performed at 200 kV for microstructure observation and for recording selected-area electron diffraction (SAED) patterns and for energy-dispersive X-ray spectroscopy (EDS) (JEM-3200FSK, JEOL, Tokyo, Japan).

X-ray photoelectron spectroscopy (XPS) (PHI5600, Physical Electronics, Chanhassen, MN, USA) was carried out to analyse the surface layer after hydrogen generation by using a magnesium X-ray anode (1253.6 eV). Binding energies were corrected by comparison with that of carbon at 284.6 eV. The surface condition of the samples was also examined by a scanning electron microscope (SEM) equipped with energy-dispersive X-ray spectroscopy (SU6600, Hitachi, Tokyo, Japan).

## 3. Results

### 3.1. Effect of Alloying Elements

**Figure 1:**
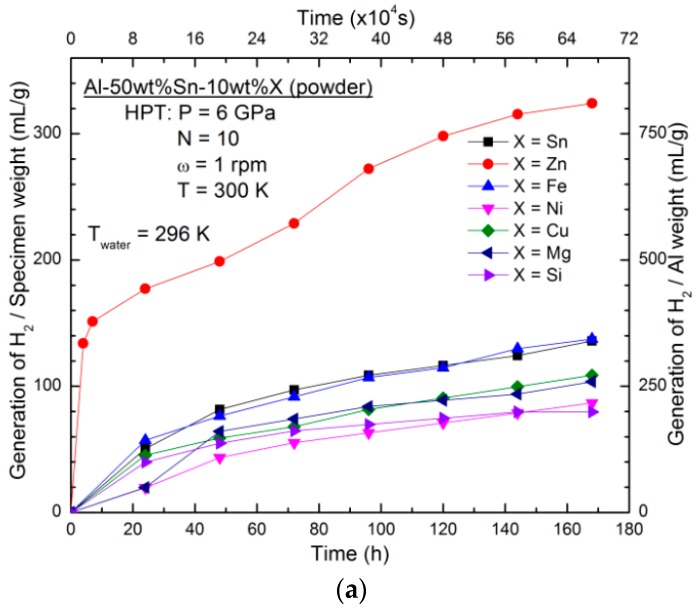

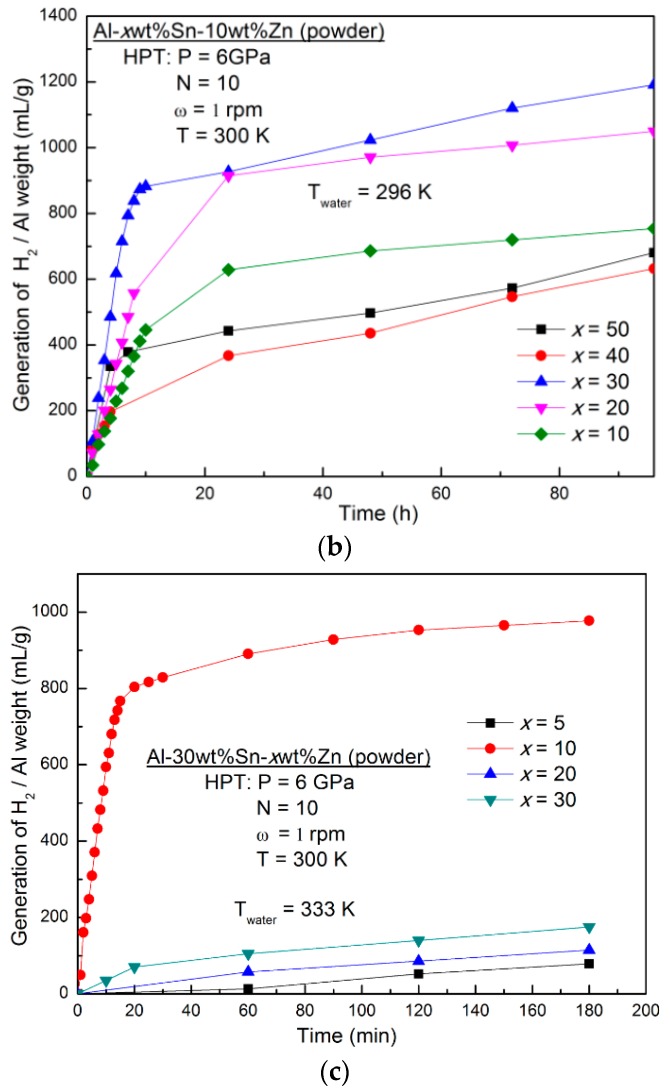
Hydrogen generation against time for (**a**) Al-50wt %Sn-10wt %X alloys (X: Sn, Zn, Fe, Ni, Cu, Mg, Si), (**b**) Al-*x*wt %Sn-10wt %Zn alloys (*x* = 10, 20, 30, 40, 50), and (**c**) Al-30wt %Sn-*x*wt %Zn alloy (*x* = 5, 10, 20, 30) processed from powders by high-pressure torsion (HPT) for *N* = 10.

Figure 1 shows the hydrogen generation performance of samples with different compositions synthesized from elemental powders using HPT for *N* = 10. Figure 1a illustrates the results of hydrogen generation from HPT-processed Al-50wt %Sn-10wt %X alloys (X: Zn, Fe, Ni, Cu, Mg, and Si) at room temperature. It is evident that the addition of Zn greatly improves the hydrogen generation speed of the binary Al–Sn alloy at room temperature, while the other alloying elements results in minor changes or a reduction in hydrogen generation.

The influence of the Sn fraction on the hydrogen generation behaviour of Al–Sn–Zn alloy is shown in Figure 1b. When the fraction of Sn increases from 10 wt % to 30 wt %, the Al–Sn–Zn alloy shows a faster hydrogen generation rate and a higher level of hydrogen production. However, with a further increase of Sn to 50 wt %, both the hydrogen generation speed and yield decrease. The sample with the addition of 30 wt % Sn gives rise to the best hydrogen generation behaviour. 

Figure 1c shows the hydrogen generation from the HPT-processed Al-30wt %Sn-*x*wt %Zn alloy (*x* = 5, 10, 20, 30). It is obvious that when the Zn fraction is 10 wt %, the sample shows significant hydrogen production. Therefore, an optimized composition of Al-30wt %Sn-10wt %Zn shows the best hydrogen generation behaviour within the selected range of materials and compositions in this study.

### 3.2. Effect of Water Temperature

**Figure 2:**
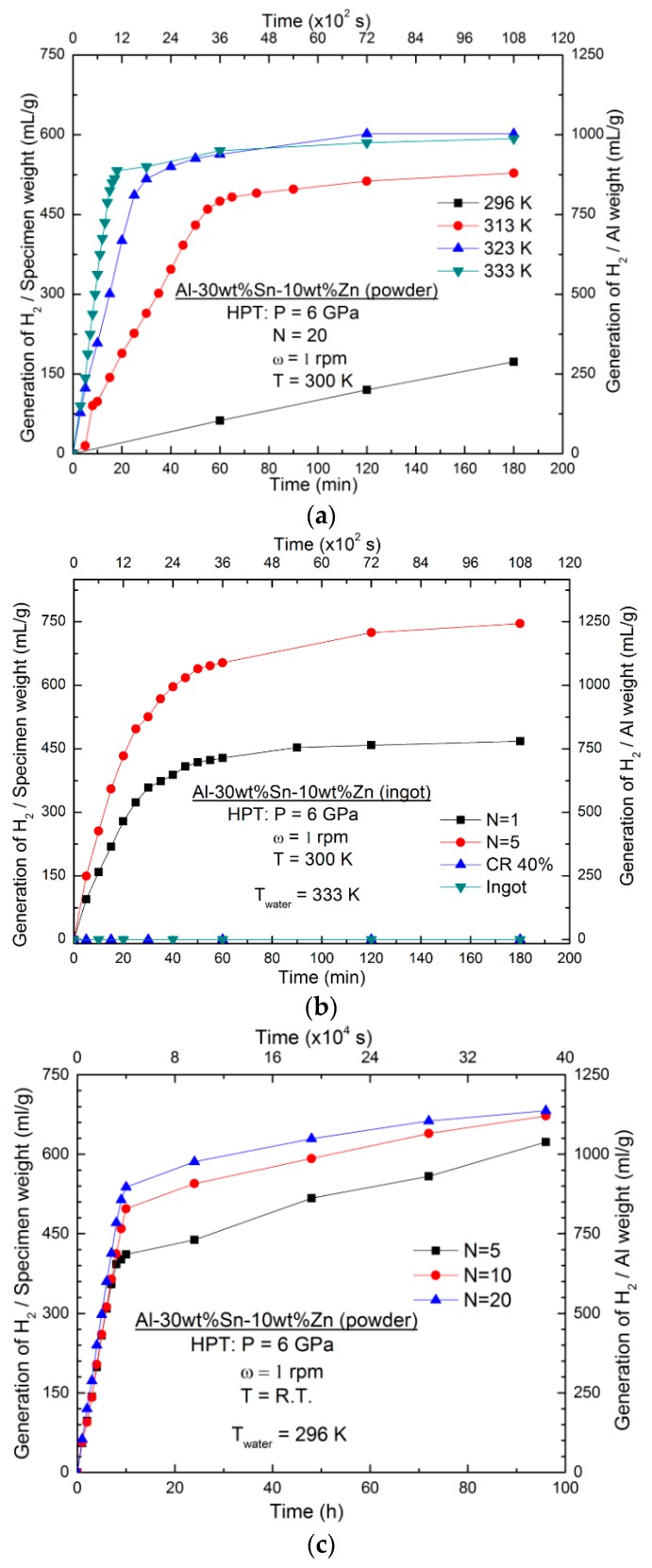
Hydrogen generation against time (**a**) at different temperatures for the Al-30wt % Sn-10wt %Zn alloy processed by HPT for *N* = 20, (**b**) at 333 K for the Al-30wt % Sn-10wt %Zn ingot processed by rolling and HPT for *N* = 1 and 5, and (**c**) at 296 K for the Al-30wt % Sn-10wt %Zn powders processed by HPT for *N* = 5, 10 and 20.

Figure 2a illustrates the temperature dependence of hydrogen generation from the Al-30wt %Sn-10wt %Zn alloy (the material with the best reaction with water) synthesized from elemental powders using HPT for *N* = 20. Both hydrogen generation rate and yield increase simultaneously with the increase in temperature. The hydrogen generation rate of the Al-30wt %Sn-10wt %Zn alloy at 333 K is as high as 90 mL min^−1^ g^−1^, which is significantly higher than the hydrogen generation rate in the binary Al–Sn alloys (maximum 50 mL min^−1^ g^−1^) [16].

### 3.3. Effect of Different Processing Routes

Figure 2b illustrates the results of hydrogen generation from the as-cast, cold-rolled, and HPT-processed Al-30wt %Sn-10wt %Zn at 333 K. While the as-cast ingot and the rolled specimen show no hydrogen generation even after 100 h, the HPT-processed samples exhibit hydrogen generation in pure water and the hydrogen generation rate and yield increase by increasing the number of HPT turns from *N* = 1 to *N* = 5 (i.e., increasing the shear strain). Figure 2c shows the results of hydrogen generation from HPT-processed Al-30wt %Sn-10wt %Zn powder samples at 296 K. It can be concluded from Figure 2 that the tendency of hydrogen generation for the HPT-processed Al-30wt %Sn-10wt %Zn alloy is consistent at different water temperatures and for samples with different initial conditions (powder or ingot). Namely, the hydrogen generation rate and yield increase with the increasing the number of HPT turns.

### 3.4. Characterization of Ingot Processed by HPT

**Figure 3:**
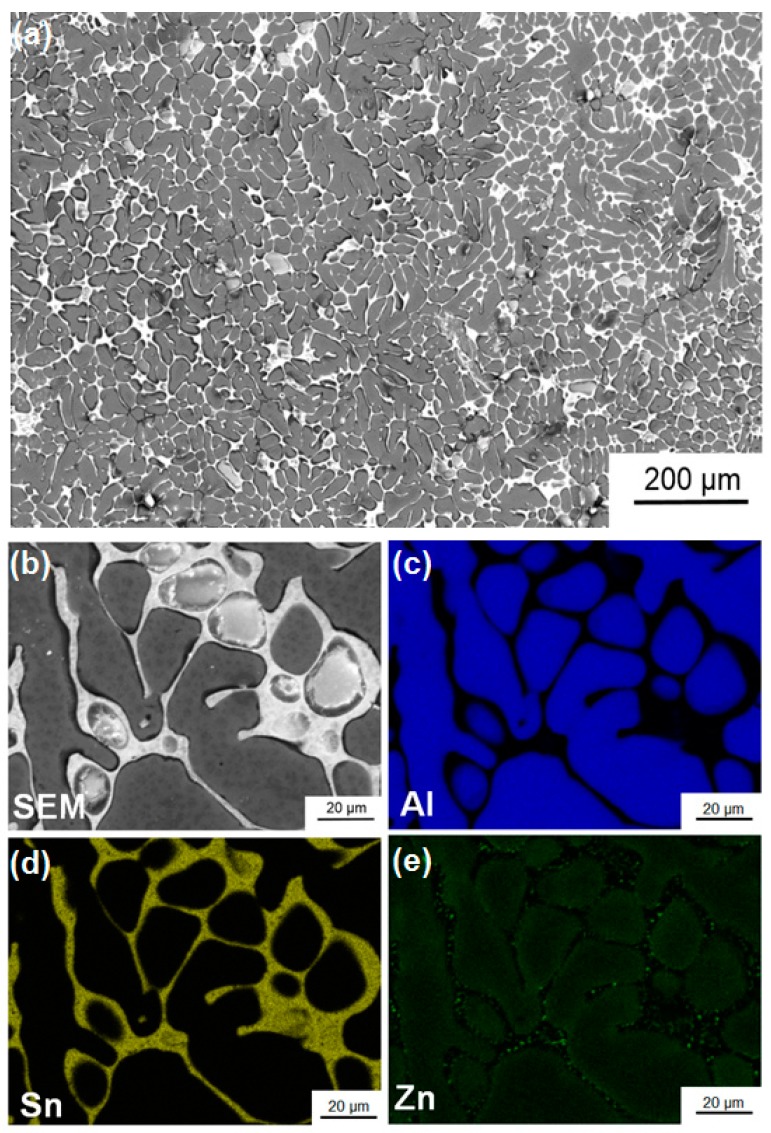
(**a**,**b**) Scanning electron microscopy (SEM) image and corresponding energy-dispersive X-ray spectroscopy (EDS) mappings with (**c**) Al, (**d**) Sn, and (**e**) Zn for the as-cast Al-30wt %Sn-10wt %Zn ingot.

**Figure 4:**
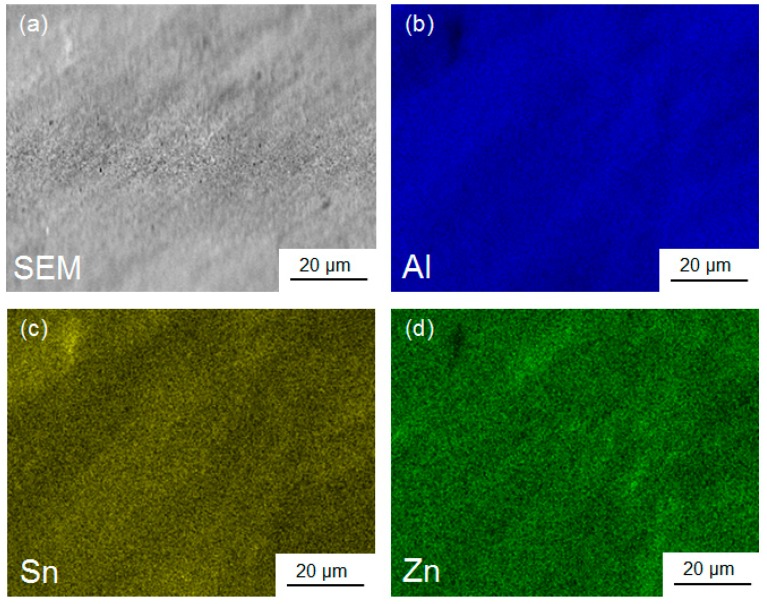
(**a**) SEM image and corresponding EDS mappings with (**b**) Al, (**c**) Sn, and (**d**) Zn for the Al-30wt %Sn-10wt %Zn ingot after processing by HPT for *N* = 5.

Figure 3a shows an SEM micrograph of the Al-30wt %Sn-10wt %Zn ingot after casting. Detailed examination of this dendritic structure by EDS analysis, as shown in Figure 3b, confirms that Sn segregates between the Al-rich dendrites, while Zn mainly presents in Al-rich dendrites and partly in Sn-rich regions. Figure 4 shows an SEM micrograph together with the EDS elemental mapping for ingot sample after HPT processing for *N* = 5. Compared with the cast sample, the distribution of the three elements becomes more homogeneous at the submicrometre level. Such a fine distribution of elements, which cannot be achieved by casting or rolling, is favourable for enhancing the fraction of nanogalvanic cells and improving the hydrogen generation behaviour.

### 3.5. Characterization of Elemental Powders Processed by HPT

**Figure 5:**
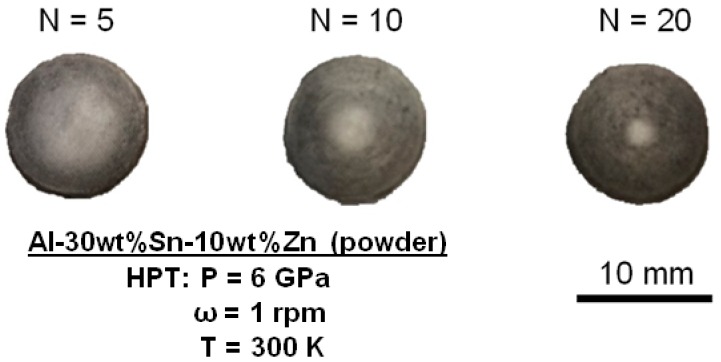
Appearance of Al-30wt % Sn-10wt %Zn discs after HPT processing of powders for *N* = 5, 10, and 20.

Since the shear strain is proportional to the distance from the disc centre in HPT processing, the microstructural features are not usually homogenous along the disc radius, but the homogeneity improves by increasing the shear strain [17,18]. The appearance of the disc samples processed from the elemental powders by HPT is shown in Figure 5. Two regions are visible on the surface of the samples: (i) a dark region which corresponds to the microstructure with a high mixing of elements and (ii) a bright region which corresponds to the microstructure with a low mixing of elements [19,20]. The dark region appears first at the edge of the disc, where the shear strain is high, while its size gradually increases with the increasing number of turns. However, even after *N* = 20 turns, there are still some small fractions of the bright region at the centre of the disc, where the shear strain is low. Therefore, one reason for the improvement of hydrogen generation by increasing the number of HPT turns can be attributed to the improvement of microstructural homogeneity.

**Figure 6:**
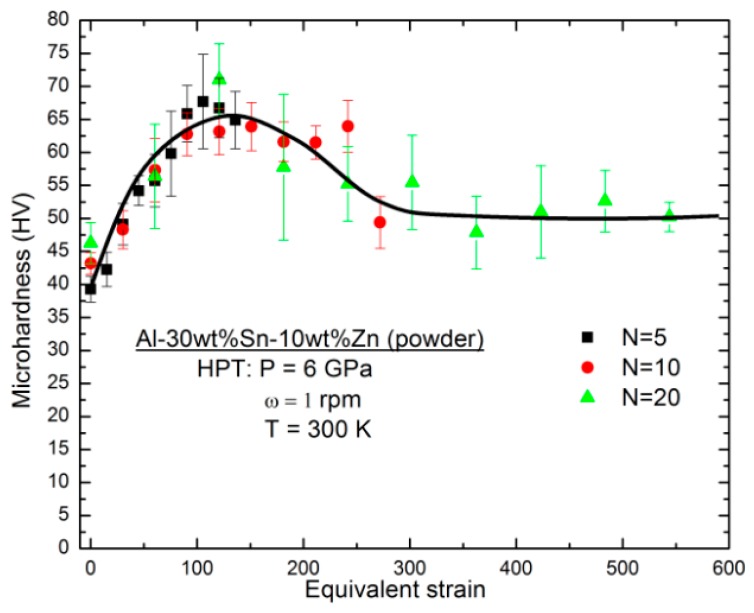
Microhardness against von Mises equivalent strain for Al-30wt %Sn-10wt %Zn samples synthesized from powders by HPT processing through *N* = 5, 10, and 20. Error bars represent standard deviation for 12 measurements.

One practical approach to examine the evolution of microstructural homogeneity in HPT-processed discs is to compare the microhardness along the disc radius [21,22,23]. Figure 6 illustrates the microhardness of HPT-processed Al-30wt %Sn-10wt %Zn powders for three different numbers of turns. The microhardness increases first with increasing the strain. However, after reaching a hardness peak, it decreases and finally saturates to a steady-state level. This hardness-strain behaviour was also reported in some low-melting-temperature metals and alloys after processing by HPT [24,25,26]. Figure 6 clearly shows that the hardness levels are not homogeneous even after HPT processing for *N* = 20, which is consistent with the appearance of the discs, as shown in Figure 5.

**Figure 7:**
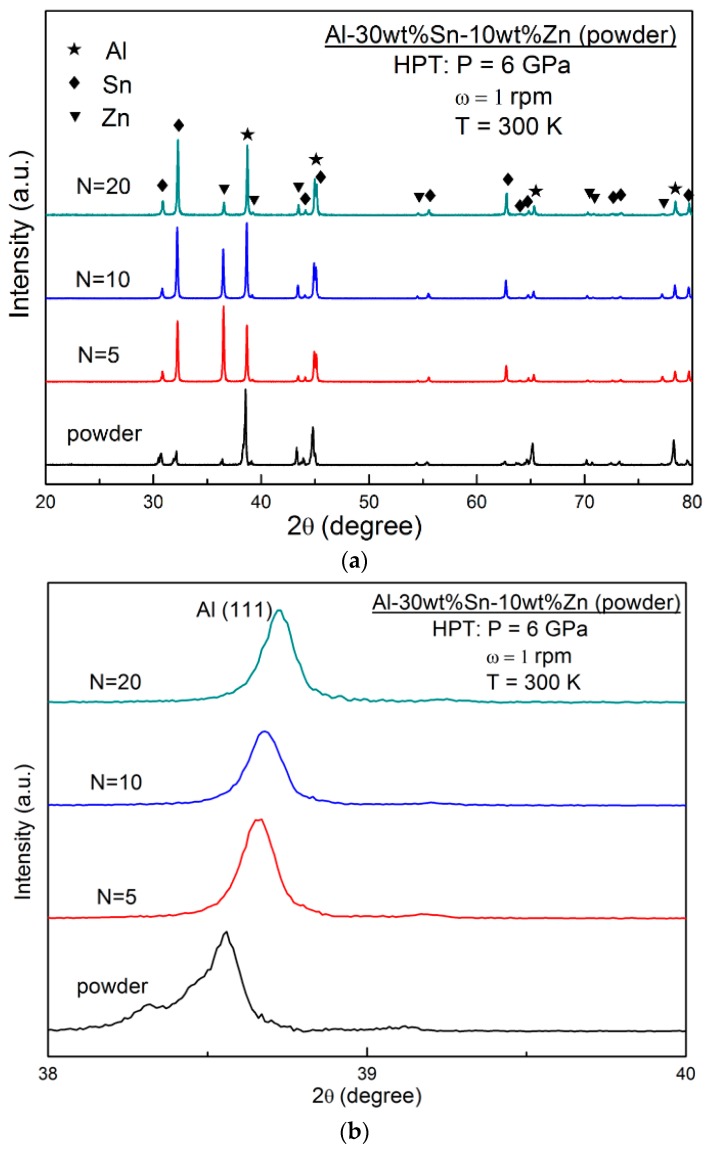
XRD profiles for (**a**) Al-30wt %Sn-10wt %Zn powders before HPT processing and after HPT processing for *N* = 5, 10, and 20 and (**b**) magnified view for Al (111) peak.

The secondary phase in Al alloys usually promotes the corrosion of matrix due to the difference of electrode potential between the matrix and the secondary phase. To check the probable effect of HPT-introduced phase transformation on hydrogen generation behaviour, XRD profiles for the powder mixture and for samples processed by HPT for *N* = 5, 10, and 20 turns were carried out, as shown in Figure 7. No new peaks were observed in Figure 7a except for the peaks of Al, Sn, and Zn, indicating that no phase transformation occurred during the HPT process. However, close examination of the profiles shows that the peak of Zn at ~36° becomes less intense by increasing the number of turns, suggesting that Zn gradually dissolves in Al- or Sn-rich regions with the increasing shear strain. This is consistent with the evidence in Figure 7b, which clearly shows the shifts of the Al (111) peak due to the changes of lattice parameters. Some previous studies of HPT-introduced phase transformation also supported this formation of Al-based solid solution after HPT [27,28].

**Figure 8:**
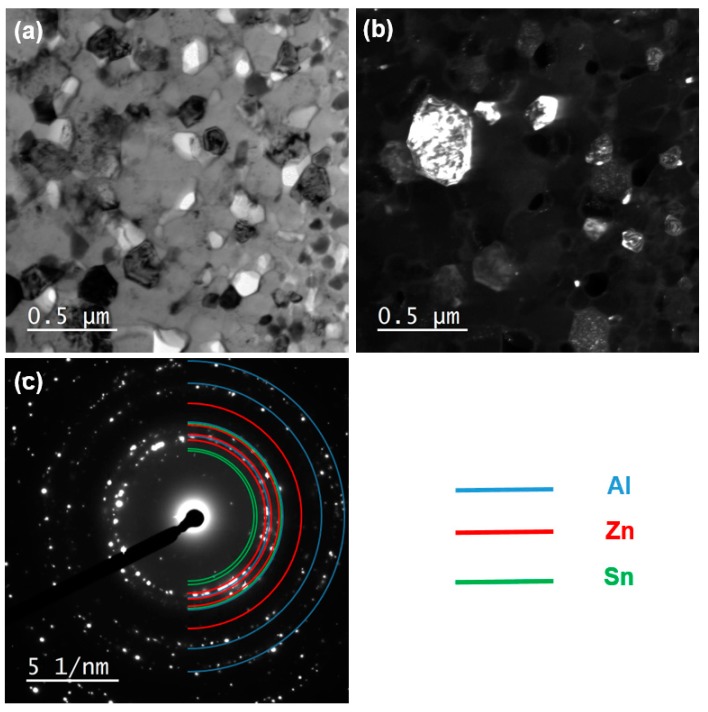
TEM (**a**) bright-field and (**b**) dark-field images including (**c**) corresponding selected-area electron diffraction (SAED) pattern for Al-30wt %Sn-10wt %Zn powders after HPT processing for *N* = 20.

**Figure 9:**
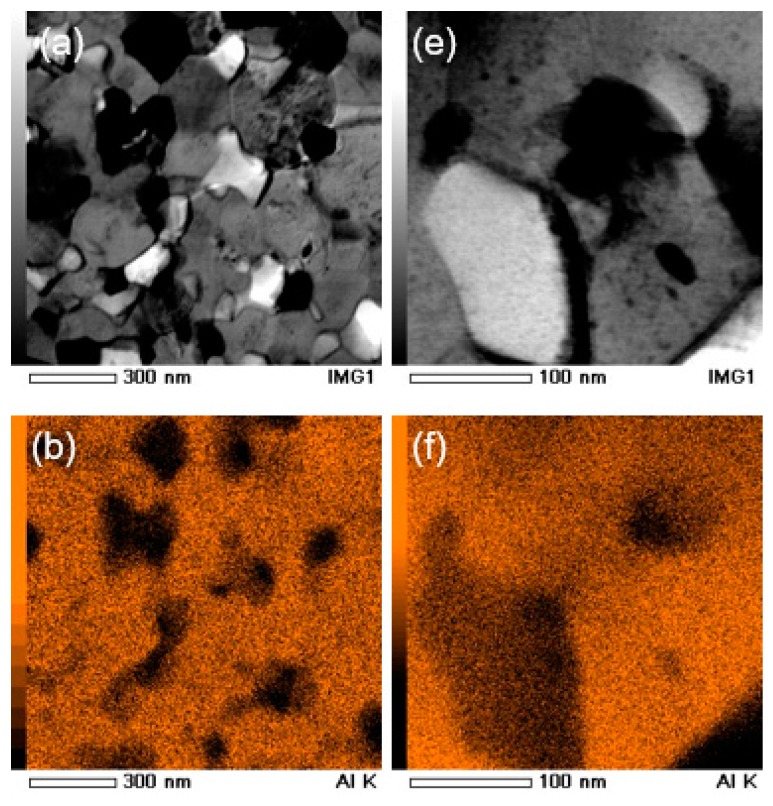

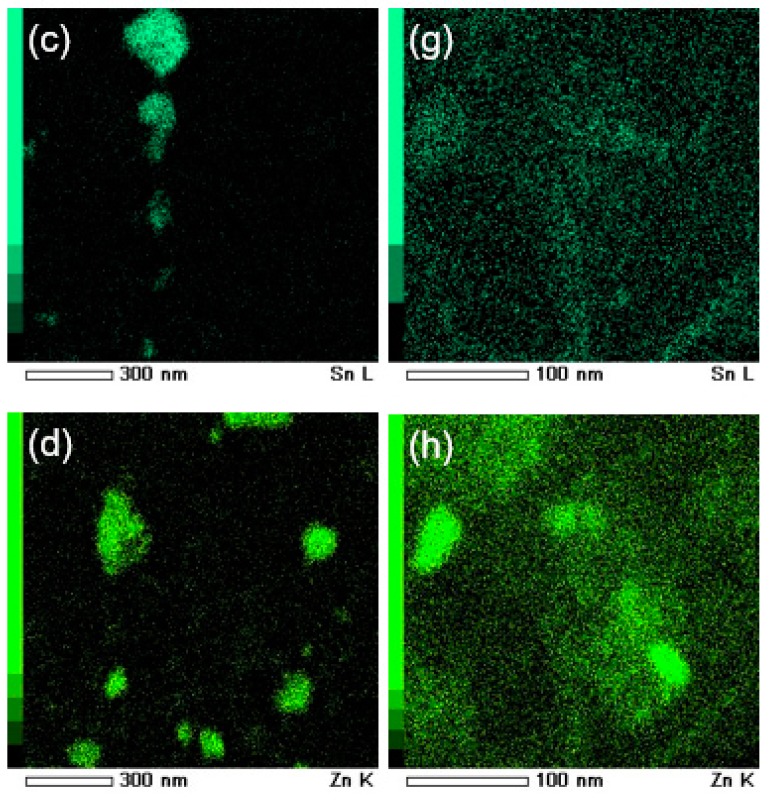
(**a**,**e**) Scanning transmission electron microscopy (STEM) bright-field image and corresponding EDS mappings with (**b**,**f**) Al, (**c**,**g**) Sn, and (**d**,**h**) Zn for Al-30wt %Sn-10wt %Zn powders after processing by HPT for *N* = 20.

Figure 8 and Figure 9 show the TEM and STEM-EDS results, respectively, for the HPT-processed Al-30wt %Sn-10wt %Zn powders after *N* = 20. Figure 8 shows that the average grain size of the alloy is ~130 nm and the diffraction spots for the three elements are visible in the SAED pattern. Figure 9 shows that the three elements are mainly present in the form of isolated ultrafine grains.

## 4. Discussion

The current results show that the hydrogen generation behaviour of Al alloys can be enhanced by not only the lattice strain and lattice defects but also by the appropriate selection of alloying additives. The addition of Fe, Ni, Cu, Mg, and Si reduces the reaction rate, but Zn can enhance the hydrogenation rate significantly. While Sn enhances the reaction rate by formation of nanogalvanic cells, the replacement of Sn by Zn cannot enhance the effect of nanogalvanic cells because Zn has a lower electrode potential when compared to Sn.

**Figure 10:**
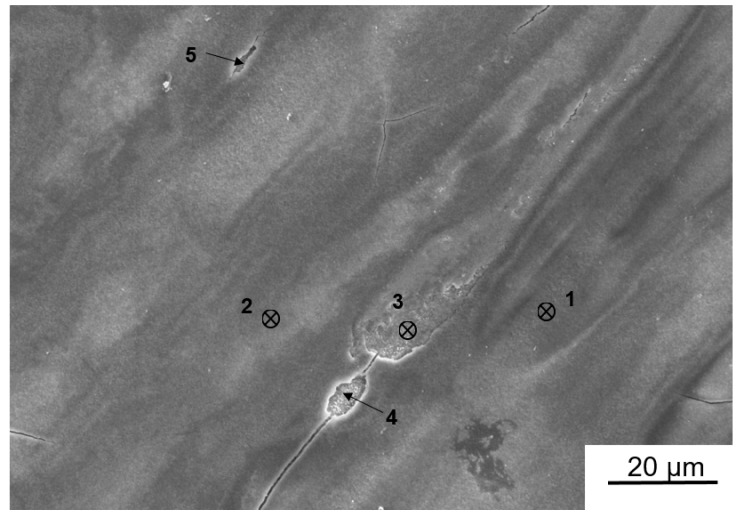
SEM micrograph taken from surface of Al-30wt %Sn-10wt %Zn processed by HPT for *N* = 20 after immersion in water for 1 min at room temperature. Numbers correspond to points for EDS analysis with results as in Table 2.

As shown in Figure 7, no phase transformation occurred after HPT processing. However, the shifts of Al peaks indicate that Zn gradually dissolved in Al by increasing the shear strain. This formation of a solid solution of Zn in Al is thought to be favourable for corrosion according to some previous studies [29,30]. A previous work carried out by Yi et al. pointed out that a slight addition of Zn to Al changed the OCP value from −1.37 V to −1.417 V [29]. Further evidence was given by Khireche et al. [30], in whose work the addition of Zn to Al shifted the potential of pitting corrosion towards more electronegative values. Therefore, in the case of the presence of Zn, corrosion will be promoted and corrosion pits will appear easily [30]. Although the formation of pits is a negative point for the corrosion resistance of Al–Zn alloys, it can have a positive impact on the hydrogen generation rate, as in this study. Figure 10 shows an SEM micrograph of the HPT-processed Al-30wt %Sn-10wt %Zn alloy after immersion into water for 1 min. After a short time of immersion, some pits can be clearly observed on the surface. Quantitative examination of the surface using SEM confirms that the average pit size is 4.9 μm, with a pitting density of 990 pit/cm^2^. Detailed EDS analysis, as summarized in Table 2, confirms that the pitting occurs in Zn-rich regions.

**Figure 11:**
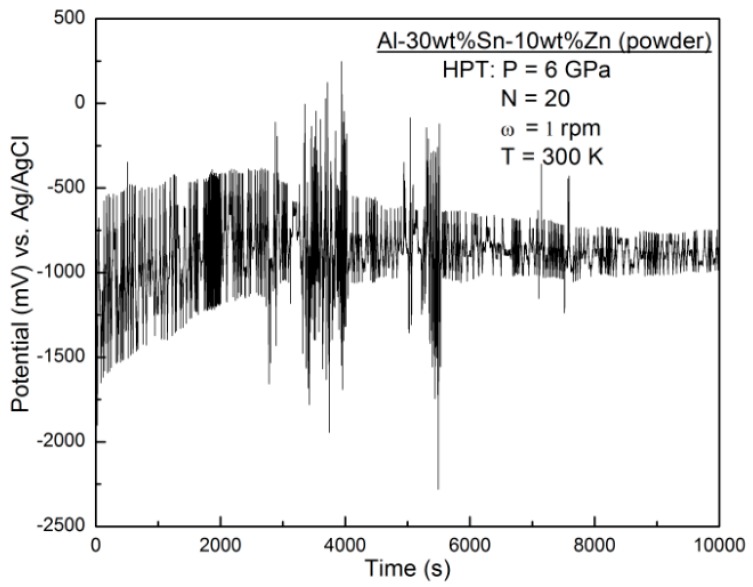
Open circuit potential (OCP) curve in water at room temperature for the Al-30wt %Sn-10wt %Zn alloy processed by HPT for *N* = 20. Potential was measured with reference to the Ag/AgCl electrode.

Figure 11 illustrates an OCP curve of the HPT-processed Al-30wt %Sn-10wt %Zn alloy in which its OCP value shows an oscillation during the immersion time. This oscillation indicates that a continuous pitting–heal process happens on the surface of the sample which finally leads to an accelerated reaction of the sample in contact with water [31,32,33,34]. When a pit forms, the potential significantly decreases to negative values and hydrogen is generated, but the potential increases again because of the healing of the surface by passivation. Formation of new pits again results in decreasing the potential and this process continues until the material completely reacts with water. From this, it can then be concluded that enhancement of pitting corrosion combined with the formation of nanogalvanic cells is effective to enhance the reaction of Al with pure water to produce hydrogen. Moreover, Zn somehow enhances the formation of cracks during the reaction due to volume expansion and increases the active surface between Al and water.

**Figure 12:**
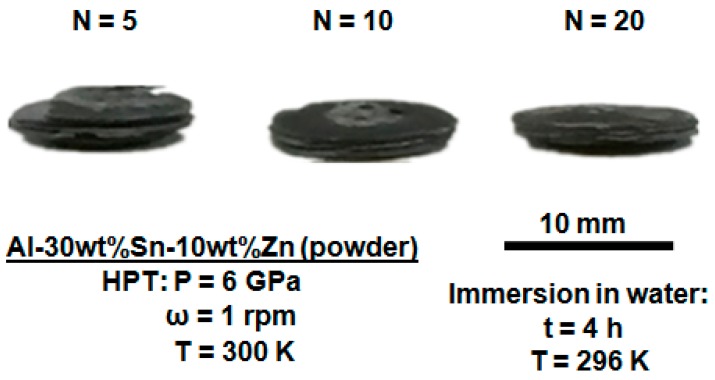
Appearance of Al-30wt %Sn-10wt %Zn discs after HPT processing of powders for *N* = 5, 10, and 20 and immersion in water at room temperature for 4 h.

**Figure 13:**
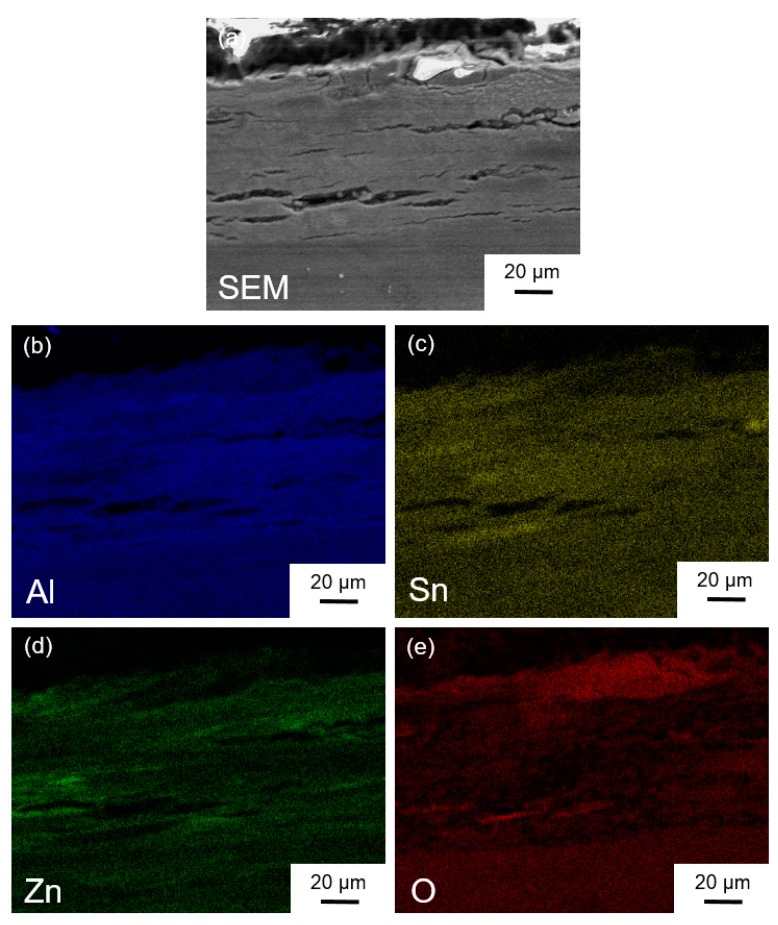
(**a**) Cross-sectional SEM image of disc and corresponding EDS mappings with (**b**) Al, (**c**) Sn, (**d**) Zn, and (**e**) O taken after HPT processing of Al-30wt %Sn-10wt %Zn powders for *N* = 20 and immersion in water at room temperature for 1 day.

The Al-30wt %Sn-10wt %Zn discs processed from powders by HPT for *N* = 5, 10, and 20 turns after immersion at room temperature in pure water for 4 h are shown in Figure 12. Despite cracking on the samples, the fragmentation rate is not so high and is incomplete even after 4 h. An SEM image and corresponding EDS mapping of the cross-section of Al-30wt %Sn-10wt %Zn powder processed by HPT for *N* = 20 after immersion at room temperature for 1 day is shown in Figure 13. It can be seen that during the immersion in water, some cracks appear on the cross-section and the surface of the discs with a crack density of 0.33 μm^2^/μm^3^ (the average distance between the cracks is 18 μm). These cracks were rarely seen after immersion of the Al–Sn samples into the water. According to literature [35,36], it is likely that the addition of Zn reduces the plasticity of the material and thus facilitates the formation of cracks. The formation of these cracks not only acts as a pathway for water but finally results in fragmentation of the sample and an increase in the active area. It is concluded that both pitting and cracking resulted from the addition of Zn to finally enhance the hydrogen generation rate in the Al–Sn–Zn system.

**Figure 14:**
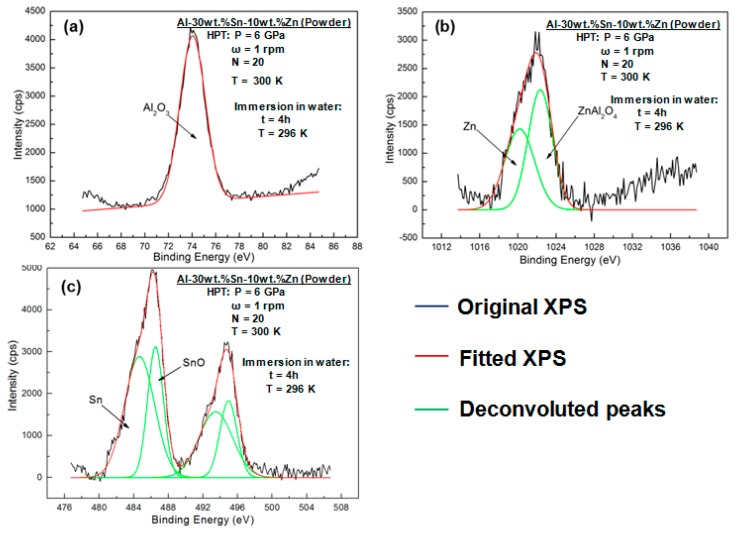
XPS spectra of (**a**) Al, (**b**) Sn, and (**c**) Zn for Al-30wt %Sn-10wt %Zn powders processed by HPT for *N* = 20 and immersed in water at room temperature for 4 h.

To investigate the mechanism for the crack formation in the Al–Sn–Zn alloys after reaction with water, XPS analysis was conducted. Figure 14 illustrates XPS spectra of powder samples processed by HPT for *N* = 20 after immersion at room temperature for 4 h. Only a peak corresponding to Al oxide can be found for the Al (2p) spectrum (Figure 14a), indicating that the main reaction product is Al_2_O_3_. For the Sn (3d_5/2_) spectrum (Figure 14b), two peaks are present at 484.2 and 486.2 eV corresponding to Sn and Sn^2+^, respectively, indicating the formation of nanogalvanic cells, in which the oxidation of Sn is suppressed in the presence of Al. The Zn (2p_3/2_) spectrum shows both Zn and Zn^2+^ species (Figure 14c). According to some earlier reports [37,38,39], Zn^2+^ corresponds to ZnAl_2_O_4_, which possesses a larger mole volume than Al_2_O_3_. The formation of ZnAl_2_O_4_ with a high mole volume can be another reason for the formation of cracks in the Al–Sn–Zn alloys after reaction with water.

**Figure 15:**
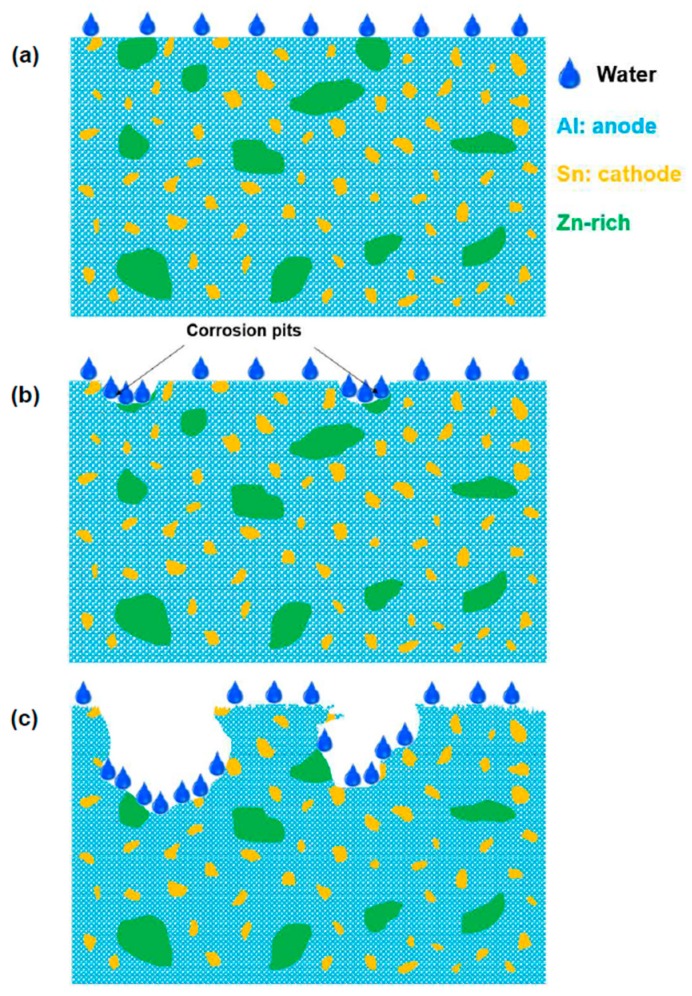
Schematic illustrations of the corrosion mechanism for the Al-30wt %Sn-10wt %Zn alloy: (**a**) immediately after immersion, (**b**) initiation of corrosion pits, and (**c**) propagation of corrosion.

Figure 15 shows schematic illustrations of the corrosion mechanism for the HPT-processed Al-30wt %Sn-10wt %Zn alloy in pure water. As shown in Figure 15a, Al/Sn nanogalvanic cells formed after HPT processing, with Al serving as the anode and Sn as the cathode. Zn presented in a form of large Zn-rich particles in some areas. When the HPT-processed alloy was put in water, as shown in Figure 15b, some corrosion pits appeared in Zn-rich areas. Such corrosion pits can allow water to go further into the material. Thus, the corrosion pits propagated and connected with each other and finally enhanced the Al hydrolysis reaction, as shown in Figure 15c.

A comparison between the hydrogen generation from the ingot, rolled sample, and HPT-processed samples with different numbers of turns confirms that increasing the shear strain plays a significant role in activation of Al–Sn–Zn alloys for hydrogen generation in water. As discussed in our previous work, the formation of nanogalvanic cells is a critical issue that should be controlled to achieve high hydrogen generation rate and yield [16]. Neither casting nor rolling is appropriate for severe mixing of elements and increasing the fraction of nanogalvanic cells. However, HPT processing results in good distribution of alloying elements at the submicrometre level or even at the nanometre level. This HPT-induced elemental mixing increases the fraction of Al/Sn nanogalvanic cells and enhances the hydrogen generation rate. Homogenous distribution of Zn by HPT processing is also beneficial for enhancing the hydrogen generation behaviour because the distribution of Zn at the submicrometre level or at the nanometre level enhances the pitting corrosion more significantly [30,31,32,34,37]. 

Based on the principle established in this study with the formation of nanogalvanic cells and pitting corrosion, an Al–Bi–C system has been successfully developed, which leads to the faster generation of hydrogen and theoretical yield, as reported elsewhere [40].

## 5. Conclusions

1. Different elements such as Zn, Fe, Ni, Cu, Mg, and Si were added to the Al–Sn alloys by HPT processing to enhance the hydrogen generation rate in contact with water. The addition of only Zn to the Al–Sn alloys improved the hydrogen generation rate and yield because of the enhancement of the pitting corrosion mechanism combined with the formation of nanogalvanic cells.
2. The changes in the amounts of Sn and Zn changed the hydrogen generation behaviour and the best reaction rate and yield were achieved for the Al-30wt %Sn-10wt %Zn alloy.
3. The Al-30wt %Sn-10wt %Zn alloy was processed by ingot casting, cold rolling, and HPT processing. While the as-cast ingot and cold-rolled samples did not exhibit hydrogen generation in water, the HPT-processed samples showed high hydrogen generation rate and yield and the hydrogen production performance was enhanced by increasing the shear strain.

## Figures and Tables

**Table 1:** Purity and mean particle size of used powders.

Element	Purity (wt %)	Size (μm)
**Al**	99.99	75
**Sn**	99.99	38
**Zn**	99.99	75
**Fe**	99.999	5
**Ni**	99.99	150
**Cu**	99.9	75
**Mg**	99.9	180
**Si**	99.999	75

**Table 2:** EDS results (in wt %) for selected areas in Figure 10. Regions 1 and 2 correspond to matrix and regions 3, 4, and 5 correspond to pits.

	O	Zn	Al	Sn
**1**	31.14	0	50.99	17.87
**2**	34.65	0	50.75	14.60
**3**	10.78	15.86	42.17	31.18
**4**	10.66	34.67	31.30	23.37
**5**	22.23	17.80	39.67	20.69

## References

[1] Mazloomi K., Gomes C. (2012). Hydrogen as an energy carrier: Prospects and challenges. Renew. Sustain. Energy Rev..

[2] Pudukudy M., Yaakob Z., Mohammad M., Narayanan B., Sopian K. (2014). Renewable hydrogen economy in Asia-Opportunities and challenges: An overview. Renew. Sustain. Energy Rev..

[3] Hwang J.J. (2012). Review on development and demonstration of hydrogen fuel cell scooters. Renew. Sustain. Energy Rev..

[4] Züttel A., Borgschulte A., Schlapbach L. (2008). Hydrogen as a Future Energy Carrier.

[5] Minić D. (2012). Hydrogen Energy-Challenges and Perspectives.

[6] Wang H.Z., Leung D.Y.C., Leung M.K.H., Ni M. (2009). A review on hydrogen production using aluminum and aluminumalloys. Renew. Sustain. Energy Rev..

[7] Elitzur S., Rosenband V., Gany A. (2014). Study of hydrogen production and storage based on aluminum-water reaction. Int. J. Hydrogen Energy.

[8] Huang X., Gao T., Pan X., Wei D., Lv C., Qin L., Huang Y. (2013). A review: Feasibility of hydrogen generation from the reaction between aluminum and water for fuel cell applications. J. Power Sources.

[9] Fan M., Xu F., Sun L. (2007). Studies on hydrogen generation characteristics of hydrolysis of the ball milling Al-based materials in pure water. Int. J. Hydrogen Energy.

[10] Ziebarth J.T., Woodall J.M., Kramer R.A., Choi G. (2011). Liquid phase-enabled reaction of Al-Ga and Al-Ga-In–Snalloys with water. Int. J. Hydrogen Energy.

[11] Wang H., Chang Y., Dong S., Lei Z., Zhu Q., Luo P., Xie Z. (2013). Investigation on hydrogen production using multicomponent aluminum alloys at mild conditions and its mechanism. Int. J. Hydrogen Energy.

[12] Ilyukhina A.V., Ilyukhin A.S., Shkolnikov E.I. (2012). Hydrogen generation from water by means of activated aluminium. Int. J. Hydrogen Energy.

[13] Mahmoodi K., Alinejad B. (2010). Enhancement of hydrogen generation rate in reaction of aluminum with water. Int. J. Hydrogen Energy.

[14] Valiev R.Z., Islamgaliev R.K., Alexandrov I.V. (2000). Bulk nanostructured materials from severe plastic deformation. Progress Mater. Sci..

[15] Valiev R.Z., Estrin Y., Horita Z., Langdon T.G., Zehetbauer M.J., Zhu Y.T. (2006). Producing bulk ultrafine-grained materials by severe plastic deformation. JOM.

[16] Zhang F., Yonemoto R., Arita M., Horita Z. (2016). Hydrogen generation from pure water using Al–Sn powders consolidated through high-pressure torsion. J. Mater. Res..

[17] Zhilyaev A.P., Nurislamova G.V., Kim B.K., Baró M.D., Szpunard J.A., Langdon T.G. (2003). Experimental parameters influencing grain refinement and microstructural evolution during high-pressure torsion. Acta Mater..

[18] Xu C., Horita Z., Langdon T.G. (2007). The evolution of homogeneity in processing by high-pressure torsion. Acta Mater..

[19] Kawasaki M., Langdon T.G. (2008). The significance of strain reversals during processing by high-pressure torsion. Mater. Sci. Eng. A.

[20] Pouryazdan M., Kaus B., Rack A., Ershov A., Hahn H. (2017). Mixing instabilities during shearing of metals. Nat. Commun..

[21] Cao Y., Wang Y.B., Figueiredo R.B., Chang L., Liao X.Z., Kawasaki M., Zheng W.L., Ringer S.P., Langdon T.G., Zhu Y.T. (2011). Three-dimensional shear-strain patterns induced by high-pressure torsion and their impact on hardness evolution. Acta Mater..

[22] Kai M., Horita Z., Langdon T.G. (2008). Developing grain refinement and superplasticity in a magnesium alloy processed by high-pressure torsion. Mater. Sci. Eng. A.

[23] Serre P., Figueiredo R.B., Gao N., Langdon T.G. (2011). Influence of strain rate on the characteristics of a magnesium alloy processed by high-pressure torsion. Mater. Sci. Eng. A.

[24] Ito Y., Horita Z. (2009). Microstructural evolution in pure aluminum processed by high-pressure torsion. Mater. Sci. Eng. A.

[25] Edalati K., Horita Z. (2011). Significance of homologous temperature in softening behavior and grain size of pure metals processed by high-pressure torsion. Mater. Sci. Eng. A.

[26] Edalati K., Yamamoto A., Horita Z., Ishihara T. (2011). High-pressure torsion of pure magnesium: Evolution of mechanical properties, microstructures and hydrogen storage capacity with equivalent strain. Scr. Mater..

[27] Mazilkin A.A., Straumal B.B., Borodachenkova M.V., Valiev R.Z., Kogtenkova O.A., Baretzky B. (2008). Gradual softening of Al–Zn alloys during high-pressure torsion. Mater. Lett..

[28] Straumal B., Kilmametov A., Ivanisenko Y., Mazilkin A., Kogtenkova O., Kurmanaeva L., Korneva A., Zieba P., Baretzky B. (2015). Phase transitions induced by severe plastic deformation: Steady-state and equifinality. Int. J. Mater. Res..

[29] Yi Y., Huo J., Wang W. (2017). Electrochemical Properties of Al–based Solid Solutions Alloyed by Element Mg, Ga, Zn and Mn under the Guide of First Principles. Fuel Cells.

[30] Khireche S., Boughrara D., Kadri A., Hamadou L., Benbrahim N. (2014). Corrosion mechanism of Al, Al-Zn and Al-Zn-Sn alloys in 3 wt. % NaCl solution. Corros. Sci..

[31] Richardson J.A., Wood G.C. (1970). A study of the pitting corrosion of Al by scanning electron microscopy. Corros. Sci..

[32] Breslin C.B., Friery L.P., Carroll W.M. (1994). The electrochemical behaviour of Al-Zn-In and Al-Zn-Hg alloys in aqueous halide solutions. Corros. Sci..

[33] El Shayeb H.A., El Wahab F.M.A., El Abedin S.Z. (2001). Electrochemical behaviour of Al, Al-Sn, Al-Zn and Al-Zn-Sn alloys in chloride solutions containing stannous ions. Corros. Sci..

[34] El Shayeb H.A., El Wahab F.M.A., El Abedin S.Z. (1999). Electrochemical behaviour of Al, A-Sn, Al-Zn and Al-Zn-Sn alloys in chloride solutions containing indium ions. J. Appl. Electrochem..

[35] Wang X., Guo M., Zhang J., Zhuang L. (2016). Effect of Zn addition on the microstructure, texture evolution and mechanical properties of Al-Mg-Si-Cu alloys. Mater. Sci. Eng. A.

[36] Shin S., Lim K., Park I. (2017). Effects of high Zn content on the microstructure and mechanical properties of Al-Zn-Cu gravity-cast alloys. Mater. Sci. Eng. A.

[37] Wu Y., Du J., Choy K., Hench L.L., Guo J. (2005). Formation of interconnected microstructural ZnAl_2_O_4_ films prepared by sol-gel method. Thin Solid Films.

[38] Strohmeier B.R., Hercules D.M. (1984). Surface spectroscopic characterization of the interaction between zinc ions and γ-alumina. J. Catal..

[39] Zhu Z., Li X., Zhao Q., Liu S., Hu X., Chen G. (2011). Facile solution synthesis and characterization of porous cubic-shaped superstructure of ZnAl_2_O_4_. Mater. Lett..

[40] Zhang F., Edalati K., Arita M., Horita Z. (2017). Fast hydrolysis and hydrogen generation on Al-Bi alloys and Al-Bi-C composites synthesized by high-pressure torsion. Int. J. Hydrogen Energy.

